# Editorial: Regulation of inflammation and metabolism in retinal neurodegenerative disorders

**DOI:** 10.3389/fnins.2022.1102385

**Published:** 2022-12-02

**Authors:** Henri Leinonen, Tianwei Ellen Zhou, Brian G. Ballios, Anu Kauppinen, Zhongjie Fu

**Affiliations:** ^1^Faculty of Health Sciences, School of Pharmacy, University of Eastern Finland, Kuopio, Finland; ^2^Department of Ophthalmology and Vision Sciences, University of Toronto, Toronto, ON, Canada; ^3^Department of Ophthalmology, Harvard Medical School, Boston Children's Hospital, Boston, MA, United States

**Keywords:** neovascularization, retina, retinopathy, metabolism, inflammation

Inflammation and metabolic alterations are two significant contributors to retinal degenerative disorders (RD) including diabetic retinopathy (DR), retinopathy of prematurity (ROP), glaucoma, and age-related macular degeneration (AMD). To facilitate research on new potential therapeutic targets, in this Research Topic we highlighted the role of inflammation and metabolism, as well as their interaction in disease progression.

## Inflammation

DR and AMD are leading causes of vision loss in working-age adults and seniors, respectively (Bressler, 2004; Lee et al., 2015). DR initially manifests with cellular features of an inflammatory phenotype and reduced retinal blood flow due to arteriopathy, leading to hypoxia. Ischemia and reperfusion (I/R) injury can be used to model the hypoxic condition and screen therapeutic approaches to inhibit DR (Zheng et al., 2007). Goit et al. showed that anti-inflammatory α-melanocyte-stimulating hormone (α-MSH) alleviated I/R-induced retinal damage in diabetic mice under hyperglycemic condition. A follow-up study in non-diabetic conditions is warranted to better understand the mechanism, and local vs. systemic effects α-MSH should be clarified.

The degeneration of retinal pigment epithelium (RPE) cells results in the death of photoreceptors and to the loss of central vision in AMD (Kauppinen et al., 2016). Wong et al. provided an update about the interplay of RPE dysfunction and the innate immune system in AMD. It has long been held that the healthy eye is an immune-privileged environment and the subretinal space is devoid of microglia/macrophages. However, following the RPE damage, circulating mononuclear phagocytes (MP) can infiltrate into the subretinal space. The accumulation of MPs is likely meant to be a defense mechanism resulting in low-grade inflammation, called para-inflammation, maintaining tissue homeostasis. However, if the normal inflammation resolution mechanism fails, for instance, due to abnormal complement function, inflammation can shift from beneficial para- to detrimental pro-inflammatory state. Accordingly, MP factors that maintain the adaptive para-inflammation in aging RPE vs. those that trigger an overt pro-inflammatory response is a promising strategy for developing therapies to slow vision loss in AMD. Certain complement pathway inhibitors have slowed down dry AMD-associated geographic atrophy progression in Phase II trials. However, the clinical risk-to-benefit profile of these approaches requires close monitoring (Lin et al., 2021; Tolentino and Tolentino, 2022). Tao et al. reviewed the role of receptor-interacting protein kinase (RIPK)-dependent necroptosis in RD. Accumulating data from experimental models suggest that RIPK inhibition can reduce inflammation and necroptosis, phenomena that enhances each other, in various types of RD, including dry AMD. More research is needed to understand whether RIPK-dependent necroptosis could be a feasible drug target in AMD and/or other types of RD.

In retinal disorders, such as AMD, RPE cells suffer from the dysfunctionality of autophagy and mitochondria both of which contribute to the induction of inflammation and disturbed cellular metabolism (Kauppinen et al., 2016). Liu et al. suggested that suppressor of cytokine signaling 2 promotes autophagy activation by regulating glycogen synthase kinase 3β and mammalian target of rapamycin. Autophagy and inflammation are known to regulate each other in RPE cells and therefore autophagy activation could reduce inflammation (Kauppinen et al., 2016). Shu et al. demonstrated that the central pro-inflammatory cytokine tumor necrosis factor-alpha (TNF-α) enhanced inflammation and disturbed glycolysis and mitochondrial morphology in primary human RPE cells, suggesting an interaction between inflammation and metabolism. They also showed that the pre-treatment of RPE cells with dimethyl fumarate (DMF) prevented TNF-α-induced mitochondrial dysfunction and morphological abnormalities. In an animal model of Parkinson's disease, DMF protected the brain by activating autophagy and modulating neuroinflammation (Lastres-Becker et al., 2016). DMF has been associated with autophagy activation in human RPE cells (Catanzaro et al., 2020).

## Metabolism

Metabolic alterations in the neural retina and the supporting cells (glia and RPE) contribute to retinal abnormalities including retinal vessel regression and proliferation (Joyal et al., 2018; Fu et al., 2019, 2021). Nilsson et al. provided an insight into the serum metabolome of extremely premature infants, which changed substantially in the neonatal period. No significant association between serum metabolome and retinopathy of prematurity and bronchopulmonary dysplasia was identified. Interestingly, circulating lipid and amino acid profile correlated with enteral energy intake. Further exploration of enteral vs. parenteral energy intake on premature complications is needed to personalize nutritional management in premature infants during the neonatal period. Shahandeh et al. summarized retinal iron metabolism and the clinical phenotypes of retinal changes including drusen-like deposits and progressive loss of visual function in iron disorders. Experimental studies also showed RPE damage and retinal dysfunction in mouse mutants with systemic iron loading and in mice with administration of exogenous iron. Further understanding of retinal pathology in systemic iron loading disorders is needed to develop therapies aimed at restoring retinal iron homeostasis. Pappenhagen et al. found that primary rat optic nerve head astrocytes had increased glycolysis under stretch stress, mimicking those findings that have been seen in glaucoma. Interestingly, the dependency of pyruvate and fatty acid as mitochondrial fuel increased in stretched optic nerve head astrocytes. Further validation in animal models *in vivo* would solidify the finding.

## Clinical features and animal models of eye diseases

The increasing recognition that animals develop retinal diseases with similar traits to humans has provided a means for testing possible treatments and successful gene therapy trials. These animal models provided much insight into the underlying pathological mechanisms. Qiang et al. summarized the clinical, multimodal imaging, and pathological features of Type 3 macular neovascularization (MNV3), described the diversity of animal models, and compared their strengths and limitations. These models have revealed the roles of retinal hypoxia, inflammation, lipid metabolism, von Hippel–Lindau (VHL)–hypoxia-inducible factor (HIF) pathway, and retinoblastoma tumor suppressor (Rb)–E2F cell cycle pathway in the development of MNV3.

Collectively, this Research Topic includes original research and review articles regarding the contribution of inflammation and metabolic alterations to retinal disorders. Improved understanding of the disease mechanisms is indispensable to the exploration of new potential therapeutictargets.

## Author contributions

All authors listed have made a substantial, direct, and intellectual contribution to the work and approved it for publication.

## Funding

This work was supported by NIH R01EY032492, NIH R01EY017017, Boston Children's Hospital (OFD/BTREC/CTREC Faculty Career Development Grant 97906, Pilot Grant 92214, and Ophthalmology Foundation 85010), Mass Lions Eye Foundation 77426 (ZF), Academy of Finland Grant 346295, Business Finland Grant 1689-31-2022, the Eye and Tissue Bank Foundation (Finland), Retina Registered Association (Finland), Sokeain Ystävät/De Blindas Vänner Registered Association (HL), and Foundation Fighting Blindness U.S. Career Development Award. CD-RM-0821-0806-UHN (BB).

## Conflict of interest

The authors declare that the research was conducted in the absence of any commercial or financial relationships that could be construed as a potential conflict of interest.

## Publisher's note

All claims expressed in this article are solely those of the authors and do not necessarily represent those of their affiliated organizations, or those of the publisher, the editors and the reviewers. Any product that may be evaluated in this article, or claim that may be made by its manufacturer, is not guaranteed or endorsed by the publisher.

## References

[B1] Bressler N. M. (2004). Age-related macular degeneration is the leading cause of blindness. JAMA 291, 1900–1901. 10.1001/jama.291.15.1900 15108691

[B2] Catanzaro M. Lanni C. Basagni F. Rosini M. Govoni S. Amadio M. (2020). Eye-light on age-related macular degeneration: targeting nrf2-pathway as a novel therapeutic strategy for retinal pigment epithelium. Front. Pharmacol. 11, 844. 10.3389/fphar.2020.00844 32581803 PMC7291861

[B3] Fu Z. Chen C. T. Cagnone G. Heckel E. Sun Y. Cakir B.. (2019). Dyslipidemia in retinal metabolic disorders. EMBO Mol. Med. 11, e10473. 10.15252/emmm.201910473 31486227 PMC6783651

[B4] Fu Z. Kern T. S. Hellstrom A. Smith L. E. H. (2021). Fatty acid oxidation and photoreceptor metabolic needs. J. Lipid. Res. 62, 100035. 10.1194/jlr.TR120000618 32094231 PMC7905050

[B5] Joyal J. S. Gantner M. L. Smith L. E. H. (2018). Retinal energy demands control vascular supply of the retina in development and disease: the role of neuronal lipid and glucose metabolism. Prog. Retin. Eye Re.s 64, 131–156. 10.1016/j.preteyeres.2017.11.002 29175509 PMC5963988

[B6] Kauppinen A. Paterno J. J. Blasiak J. Salminen A. Kaarniranta K. (2016). Inflammation and its role in age-related macular degeneration. Cell Mol. Life Sci. 73, 1765–1786. 10.1007/s00018-016-2147-8 26852158 PMC4819943

[B7] Lastres-Becker I. Garcia-Yague A. J. Scannevin R. H. Casarejos M. J. Kugler S. Rabano A.. (2016). Repurposing the NRF2 activator dimethyl fumarate as therapy against synucleinopathy in Parkinson's disease. Antioxid. Redox Signal. 25, 61–77. 10.1089/ars.2015.6549 27009601 PMC4943471

[B8] Lee R. Wong T. Y. Sabanayagam C. (2015). Epidemiology of diabetic retinopathy, diabetic macular edema and related vision loss. Eye Vis. (Lond). 2, 17. 10.1186/s40662-015-0026-2 26605370 PMC4657234

[B9] Lin J. B. Halawa O. A. Miller J. W. Vavvas D. G. (2021). Complement inhibition for geographic atrophy: a tempting target with mixed results. J. Clin. Med. 10. 10.3390/jcm10132890 34209660 PMC8267692

[B10] Tolentino M. J. Tolentino A. J. (2022). Investigational drugs in clinical trials for macular degeneration. Expert. Opin. Investig. Drugs. 31, 1067–1085. 10.1080/13543784.2022.2113375 35962560

[B11] Zheng L. Gong B. Hatala D. A. Kern T. S. (2007). Retinal ischemia and reperfusion causes capillary degeneration: similarities to diabetes. Invest Ophthalmol. Vis. Sci. 48, 361– 10.1167/iovs.06-0510 17197555

